# Some *Gammaproteobacteria* are enriched within CD14^+^ macrophages from intestinal lamina propria of Crohn’s disease patients versus mucus

**DOI:** 10.1038/s41598-020-59937-w

**Published:** 2020-02-19

**Authors:** Yuki Sekido, Junichi Nishimura, Kazuhiro Nakano, Takeaki Osu, Cheryl-Emiliane T. Chow, Hiroshi Matsuno, Takayuki Ogino, Shiki Fujino, Norikatsu Miyoshi, Hidekazu Takahashi, Mamoru Uemura, Chu Matsuda, Hisako Kayama, Masaki Mori, Yuichiro Doki, Kiyoshi Takeda, Motoi Uchino, Hiroki Ikeuchi, Tsunekazu Mizushima

**Affiliations:** 1 Department of Gastroenterological Surgery, Graduate School of Medicine, Osaka University, Suita, Japan; 2 Department of Gastroenterological Surgery, Osaka International Cancer Institute, Osaka, Japan; 3 Research Institute, EA Pharma Co., Ltd., Tokyo, Japan; 4 Second Genome, Inc., San Francisco, California USA; 5 Laboratory of Immune Regulation, Department of Microbiology and Immunology, Graduate School of Medicine, Osaka University, Suita, Japan; 6 Laboratory of Mucosal Immunology, Immunology Frontier Research Center, Osaka University, Suita, Japan; 7 Department of Surgery and Science, Graduate School of Medical Science, Kyushu University, Fukuoka, Japan; 8 Department of Inflammatory Bowel Disease, Division of surgery, Hyogo College of Medicine, Hyogo, Japan; 9 Department of Therapeutics for Inflammatory Bowel Diseases, Graduate School of Medicine, Osaka University, Suita, Japan

**Keywords:** Bacterial genes, Crohn's disease, Innate immunity, Microbiome, Crohn's disease

## Abstract

Crohn’s disease causes chronic inflammation in the gastrointestinal tract and its pathogenesis remains unclear. In the intestine of Crohn’s disease patients, CD14^+^CD11^+^CD163^low^ macrophages contribute to inflammation through the induction of Th17 cells and production of inflammatory cytokines; the CD14^+^CD11c^+^163^high^ fraction is anti-inflammatory through the production of IL-10 in normal cases. In this report, the 16S rRNA gene amplicon sequencing method was used to identify bacteria that are specifically present in intestinal CD14^+^CD11c^+^ macrophages of Crohn’s disease patients. Bacteria present in intestinal CD14^+^CD11c^+^ macrophages and mucus of Crohn’s disease patients were separated into different clusters in principal coordinates analysis. There was a statistically significant increase in the relative composition of CD14^+^CD11c^+^ macrophages from mucus in two phyla (*Proteobacteria* [p = 0.01] and *Actinobacteria* [p = 0.02]) and two families (*Moraxellaceae* [p < 0.001] and *Pseudomonadaceae* [p = 0.01]). In addition, OTU-1: *Acinetobacter* and OTU-8: *Pseudomonadaceae* tended to concentrate in the CD14^+^CD11c^+^CD163^low^ subset, whereas OTU-10: *Proteus*, OTU-15: *Collinsella* tended to concentrate more in the CD14^+^CD11c^+^CD163^high^ subset than the other subset and mucus.

## Introduction

Crohn’s disease causes chronic inflammation in the gastrointestinal tract and is known as inflammatory bowel disease (IBD) together with ulcerative colitis, yet fundamental therapy has not yet been established. Inflammatory reactions are usually controlled in the intestinal tract and intestinal homeostasis is maintained. In IBD, chronic inflammation is thought to occur by maintaining an excessively activated state of immune cells through the involvement of several factors, including genetic factors, environmental factors, immune abnormality, and intestinal bacteria^1,2^.

In the intestinal tract of Crohn’s disease, CD14^+^ macrophages are increased compared to normal intestine. CD14^+^ macrophages are more ability to produce inflammatory cytokines such as IL-23, TNF-α, and IL-6 than CD14^−^ macrophages^3^. CD14^+^ macrophages also have the ability to induce Th17 cells, but in the intestines of Crohn’s disease, the inducibility of Th17 cells is accelerated in the presence of bacteria-derived antigen^4^. We have shown that CD14^+^CD11c^+^ macrophages can be divided into populations that express CD163 at low and high levels. The CD14^+^CD11c^+^CD163^low^ fraction of normal intestine induces differentiation of Th17 cells; in Crohn’s disease, this induction capability has been accelerated by increased production of inflammatory cytokines^5^. In contrast, the CD14^+^CD11c^+^163^high^ fraction is anti-inflammatory through the production of IL-10 in normal cases^6^.

Macrophages, which are innate immune cells, phagocytose pathogenic microorganisms; however, there are some reports that certain strains from Crohn’s disease patients survived without being digested in the phagosomes of human monocyte-derived macrophages^7,8^. In addition, it has also been reported that TNF-α production was enhanced in a macrophage cell line infected with *Escherichia coli* isolated from Crohn’s disease patients^9^. These reports suggest that some bacteria from Crohn’s disease patients may survive in the host intestinal macrophages and activate an inflammatory response.

In the last several years, next generation sequencing has made it possible to comprehensively analyze the human microbiome and many analyses of bacterial flora in faeces and mucosal tissues of Crohn’s disease patients have been conducted^10–14^. However, there is limited knowledge about the bacterial flora present in macrophages, and in particular regarding the microbiota in macrophages from the intestinal lamina propria of Crohn’s disease patients.

In this study, we illuminated the intracellular microbiota in CD14^+^CD11c^+^ macrophages from the intestinal lamina propria using the 16S rRNA gene amplicon sequencing method to identify bacteria that are specifically present in intestinal CD14^+^CD11c^+^ macrophages of Crohn’s disease patients, and compared bacterial flora between CD14^+^CD11c^+^CD163^low^ and CD14^+^CD11c^+^CD163^high^ macrophages, that have opposite inflammatory functions, to investigate whether there is a difference in bacterial flora.

## Materials and Methods

### Intestinal samples

Intestinal samples were obtained through ileum resection at Hyogo College of Medicine Hospital from patients with a confirmed diagnosis of Crohn’s disease endoscopically, radiographically, or histologically. Cases that were difficult to distinguish from other disease such as ulcerative colitis or Behçet’s disease were excluded. Parts of the samples with macroscopically and histologically inflammatory finding unique to Crohn’s disease were used.

This was a clinical study in which Osaka University received samples from Hyogo College of Medicine Hospital with. All samples were obtained with written informed consent according to the principles of the declaration of Helsinki after approval by the ethics committee of the Osaka University Medical Hospital (Approval number: 10261).

### Isolation of lamina propria cells and sorting CD14^+^CD11c^+^ macrophage subsets

Cells in the lamina propria were isolated from intestinal samples and the CD14^+^CD11c^+^CD163^low^ subset and CD14^+^CD11c^+^CD163^high^ subset were sorted using flow cytometry as reported previously^15^. In brief, resected intestinal samples were washed in PBS to remove feces. The mucus surface was wiped with a scalpel blade, and the exposed lower mucus was again wiped with a new scalpel blade to obtain mucus samples. The intestinal samples after wiping off all the remaining mucus were put into HBSS containing 5 mM EDTA and incubated for 6 min under shaking conditions. After removing muscle layer with Cooper scissors, the mucosal layer was cut into small pieces and incubated in RPMI 1640 containing 4% fetal bovine serum (FBS), 1 mg/ml collagenase D (Roche, Basel, Switzerland), 1 mg/ml dispase (Invitrogen, Carlsbad, CA), and 80 U/ml DNase I (Roche) for 60 min in a 37 °C shaking water bath. The digested tissues were resuspended with HBSS containing 5 mM EDTA and passed through a 40 μm cell strainer. The isolated cells were resuspended in 7 ml of 20% Percoll and 2 ml of 40% Percoll in a 15 ml tube. Percoll gradient separation was performed at 500 × g for 30 min at 4 °C. The LPCs were collected at the interface of the Percoll gradient and washed with PBS containing 4% FBS.

The cell suspension was stained with Fixable Viability Stain 450 (BD Horizon). After washing with PBS containing 4% FBS, these cells were stained with the lineage markers (FITC-conjugated anti-human CD3 (HIT3a, BioLegend), CD19 (HIB19, BioLegend), CD20 (2H7, BioLegend), CD56 (HCD56, BioLegend), PE/Cy7-conjugated anti-human HLA-DR (L243, BioLegend), PE-conjugated anti-human CD14 (HCD14, BioLegend), APC-conjugated anti-human CD11c (B-ly6, BD Biosciences), and PerCP/Cy5.5-conjugated anti-human CD163 (GHI/61 BioLegend). Flow cytometry was performed using a FACS Aria II system (BD Biosciences) to collect CD14^+^CD11c^+^CD163^low^ subset and CD14^+^CD11c^+^CD163^high^ subset. The sorted cell suspension and mucus samples collected in advance were lysed in BufferT1 (NucleoSpin Tissue XS, MACHEREY-NAGEL) and incubated 10 min at 95 °C, then stored at −80 °C.

### DNA extraction

DNA was extracted using the NucleoSpin Tissue XS (MACHERRY-NAGEL) kit according to the kit protocol, with the modification of adding 90 mg lysozyme (L6876, Sigma-Aldrich) and 12000 U achromopeptidase (TBL-1, Wako Pure Chemical) and incubating 90 min at 37 °C prior to the kit-specified proteinase K treatment of 30 min at 56 °C.

### 16S rRNA gene amplicon sequencing

The 16S rRNA V4 region of the DNA samples were PCR amplified using the 515-F and 806-R custom bar-coded fusion primers that target the V4 region and include the sequencing index barcodes, as described in Caporaso *et al*.^16^. Due to low microbial biomass available, samples were subjected to two rounds of PCR. PCR conditions were modified from Caporaso *et al*.^16^ as follows: 25 µl of Q5 Hot Start High-Fidelity 2X Master Mix, 2.5 µl of 10 µM 515-F primer, 2.5 µl of 10 µl 806-R primer and 20 ng of input nucleic acids in a 50 µl total reaction volume. Cycling conditions were 1 cycle of 98 °C for 30 seconds, 30 cycles of 98 °C at 10 sec, 50 °C for 30 seconds, and 72 °c for 30 seconds, followed by 1 cycle of 72 °C for 10 min and then held at 4 °C. The first PCR used 20 ng of extracted DNA as measured by fluorometic analysis using Quant-iT Picogreen (ThermoFisher), and the second PCR used 20 ng of the purified product from the first PCR round. PCR products were purified using magnetic beads (Ampure XP) and quantified by Quant-iT Picogreen. All libraries were pooled equimolar and sequenced using 2 × 250 PE cycles on an Illumina MiSeq with custom sequencing primers, as described^16^.

### Data analysis

Only the samples that passed a minimum read depth of 3,000 were included in downstream statistical analyses.

Sequenced reads were merged with USEARCH, and the combined sequences were compared with an in-house database. All sequences that hit a unique strain with an identity of ≥99% were assigned a strain-level operational taxonomic unit (OTU). An identity difference of ≥0.25% between the best and second-best hit was needed to ensure specificity of the strain hits. To calculate strain abundances, all sequences were mapped against the representative sequence from each strain-level OTU. After sequences that were not matched to any strain were quality filtered and dereplicated, the resulting sequences were clustered at 97% by the UPARSE clustering algorithm^17^ for filtered chimeric OTUs, and a representative sequence per OTU was determined. The non-strain sequences that passed the filtering were then mapped to the representative sequences for *de novo* OTUs, and an abundance table for all strain and *de novo* OTUs was generated. The representative OTU sequences were also assigned an additional taxonomic classification using mothur’s Bayesian classifier, which was trained against the Greengenes database (version 2013.5)^18^.

To evaluate alpha diversity, the OTU richness and the Shannon diversity index were obtained. In the assessment of beta diversity, abundance-weighted sample pairwise differences were calculated with the Bray-Curtis dissimilarity. The binary dissimilarity values were calculated using the Jaccard index. Principal Coordinates Analysis, a plotting method used to describe two-dimensional ordination, was used to assess the inter-sample relationships and dendrogram-style hierarchical clustering of the samples were created. Evaluation of microbiome beta diversity was tested by the Permutational Analysis of Variance (PERMANOVA) method of the Bray-Curtis dissimilarity matrix^19^. The univariate differential abundance of OTUs was tested using a negative binomial noise model for the overdispersion and Poisson process intrinsic to this data.

16S rRNA gene sequencing and microbiome data analysis were provided by Second Genome, Inc.

## Results

Twelve patients were included in this study: 9 males and 3 females. Median age at the operation were 45.5 (range 20–67) years old. Twelve macrophage samples from the CD14^+^CD11c^+^CD163^low^ fraction, 11 macrophage samples from the CD14^+^CD11c^+^CD163^high^ fraction, and 12 mucus samples met the read depth criteria mentioned above.

For the library size of the sample, the mean sequencing depth of the CD14^+^CD11c^+^CD163^low^ subset was 24,674 reads (range, 4,602–88,780), that of CD14^+^CD11c^+^CD163^high^ subset was 23,721 reads (range, 3,157–124,938), and that of mucus was 46,918 reads (range, 4,613–115,322).

### Diversity and similarity of bacteria present in CD14^+^CD11c^+^ macrophages of the lamina propria and mucus from Crohn’s disease patients

**Figure 1:**
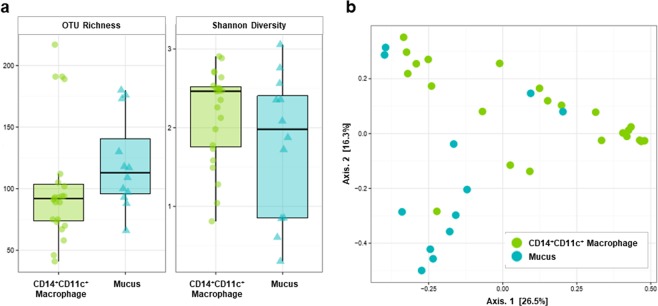
Diversity and similarity of bacteria present in CD14^+^CD11c^+^ macrophage of lamina propria and mucus from Crohn’s disease patients. (**a**) The OTU richness and Shannon Diversity Index of bacteria present in CD14^+^CD11c^+^ macrophage and mucus. OTU richness represents the number of OTUs in each sample. The Shannon Diversity Index takes into account the richness and evenness of OTUs within samples. (**b**) Weighted principal coordinates analysis of microbiome samples of CD14^+^CD11c^+^ macrophage and mucus. This analysis uses the sample-to-sample dissimilarity values to position the points relative to each other by maximizing the linear correlation between the dissimilarity values and the plot distances.

Alpha diversity of bacteria presented in CD14^+^CD11c^+^ macrophages and mucus in the intestinal mucosal lamina propria of Crohn’s disease patients was evaluated (Fig. 1a). The mean number of OTUs in CD14^+^CD11c^+^ macrophages and mucus were 102 (SD 48.1) and 121 (SD 37.4), respectively, meaning that the species richness tended to decrease from mucus to CD14^+^CD11c^+^ macrophages (p = 0.08). In contrast, the Shannon Diversity Index, which considers species abundance and species uniformity, showed no tendency to decrease with CD14^+^CD11c^+^ macrophages at 2.16 (SD 0.592) and mucus at 1.78 (SD 0.917) (p = 0.25). Therefore, it was suggested that bacteria in macrophages tended to decrease monopolistic OTUs and increase evenness compared with bacteria in mucus.

Next, weighted principal coordinates analysis was performed to evaluate the homology of each sample (Fig. 1b). Samples were separated into different clusters in general by CD14^+^CD11c^+^ macrophages and mucus by Axis 2, indicating that the similarity of the bacterial flora differed between mucus and CD14^+^CD11c^+^ macrophages.

### Bacteria concentrated in CD14^+^CD11c^+^ macrophages present in the mucosal lamina propria of Crohn’s disease patients

**Figure 2:**
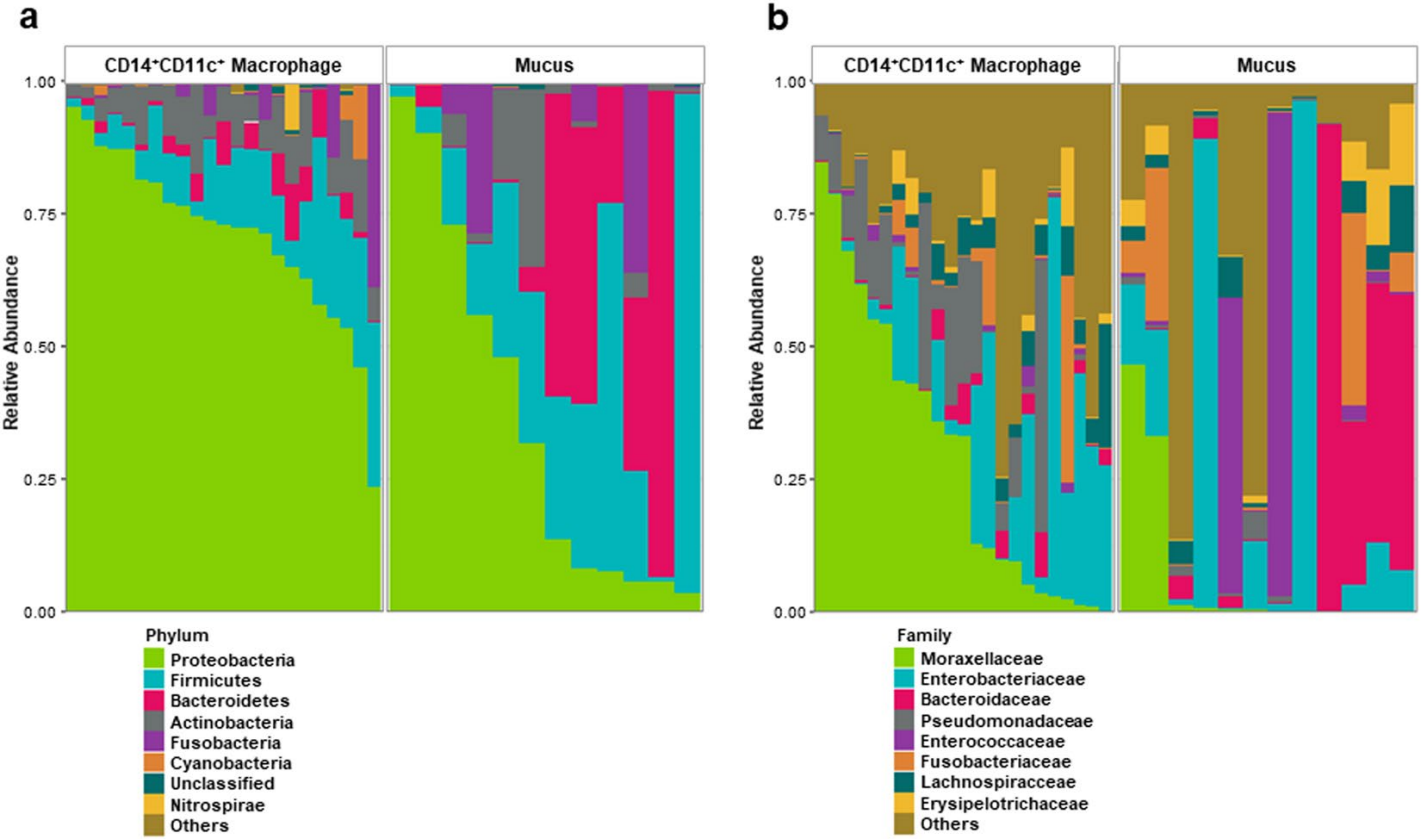
Composition of the microbiome present in CD14^+^CD11c^+^ macrophage of lamina propria and mucus from Crohn’s disease patients. (**a**) Composition at the phylum level. (**b**) Composition at the family level. The relative abundance ratios of each microorganism group are shown in different colors.

16S rRNA sequencing was performed to clarify the bacterial composition of the bacterial flora in CD14^+^CD11c^+^ macrophages and mucus at the levels of Phylum and Family (Fig. 2). There was a statistically significant increase in the relative composition of CD14^+^CD11c^+^ macrophages from mucus in two phyla: *Proteobacteria* (p = 0.01) and *Actinobacteria* (p = 0.02) (Fig. 2a, Table 1). Also, the families *Moraxellaceae* (p < 0.001) and *Pseudomonadaceae* (p = 0.01) had statistically significant increases in composition ratio in CD14^+^CD11c^+^ macrophages compared to mucus (Fig. 2b, Table 2).

**Table 1:** The eight most abundant taxa at the Phylum level.

Phylum	Chi-square	KW p-value	CD14^+^CD11c^+^ Macrophage Mean % (SD)		Mucus Mean % (SD)	
***Proteobacteria***	**6**.**9758**	**0**.**01**	**71**.**0**	**(16**.**3)**	**36**.**7**	**(35**.**1)**
*Firmicutes*	2.6679	0.10	12.8	(8.84)	28.4	(27.7)
*Bacteroidetes*	1.5652	0.21	3.06	(3.42)	22.0	(30.3)
***Actinobacteria***	**5**.**5845**	**0**.**02**	**7**.**74**	**(4**.**29)**	**5**.**93**	**(9**.**85)**
*Fusobacteria*	0.0109	0.92	3.21	(8.49)	6.6	(12.5)
*Cyanobacteria*	3.4507	0.06	0.961	(3.03)	0.00101	(0.00163)
*unclassified*	1.0157	0.31	0.504	(0.481)	0.359	(0.422)
*Nitrospirae*	0.0046	0.95	0.412	(1.93)	0.00593	(0.011)

KW, Kruskal-Wallis rank sum test; SD, standard deviation. Bold letters indicate a statistically significant increase in the relative composition of CD14^+^CD11c^+^ macrophage from mucus.

**Table 2:** The eight most abundant taxa at the Family level.

Family	Chi-square	KW p-value	CD14^+^CD11c^+^ Macrophage Mean % (SD)		Mucus Mean % (SD)	
***Moraxellaceae***	**12**.**5652**	**<0**.**001**	**30**.**2**	**(27**.**3)**	**6**.**94**	**(15**.**6)**
*Enterobacteriaceae*	0.0048	0.94	16.9	(19.2)	21.8	(33.6)
*Bacteroidaceae*	1.9324	0.16	1.94	(2.59)	19.5	(30.0)
***Pseudomonadaceae***	**7**.**1606**	**0**.**01**	**11**.**2**	**(13**.**3)**	**1**.**04**	**(1**.**41)**
*Enterococcaceae*	1.8395	0.18	0.676	(1.08)	12.9	(29.3)
*Fusobacteriaceae*	0.0435	0.83	3.21	(8.49)	6.6	(12.5)
*Lachnospiraceae*	0.0193	0.89	4.26	(4.98)	3.59	(3.80)
*Erysipelotrichaceae*	0.3913	0.53	2.05	(3.75)	4.11	(5.59)

KW, Kruskal-Wallis rank sum test; SD, standard deviation. Bold letters indicate a statistically significant increase in the relative composition of CD14^+^CD11c^+^ macrophage from mucus.

### Diversity and similarity of bacteria present in CD163^low^ and CD163^high^ subsets of CD14^+^CD11c^+^ macrophages from the mucosal lamina propria of Crohn’s disease patients

**Figure 3:**
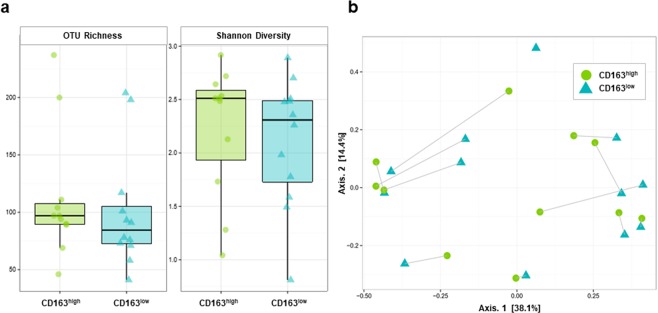
Diversity and similarity of bacteria present in CD14^+^CD11c^+^CD163^low^ and CD14^+^CD11c^+^CD163^high^ macrophage of lamina propria from Crohn’s disease patients. (**a**) The OTU richness and Shannon Diversity Index of bacteria present in CD14^+^CD11c^+^CD163^low^ and CD14^+^CD11c^+^CD163^high^ macrophage. OTU richness represents the number of OTUs in each sample. The Shannon Diversity Index takes into account the richness and evenness of OTUs within samples. (**b**) Weighted principal coordinates analysis of microbiome samples of CD14^+^CD11c^+^CD163^low^ and CD14^+^CD11c^+^CD163^high^ macrophage. This analysis uses the sample-to-sample dissimilarity values to position the points relative to each other by maximizing the linear correlation between the dissimilarity values and the plot distances.

Comparison of alpha diversity of bacteria present in macrophages from the CD14^+^CD11c^+^CD163^low^ and CD14^+^CD11c^+^CD163^high^ fractions (Fig. 3a). Between the CD14^+^CD11c^+^CD163^low^ fraction and the CD14^+^CD11c^+^CD163^high^ fraction, the mean number of OTUs were 100 (SD 5.1) and 112 (SD 56.1), respectively; the difference was not statistically significant (p = 0.39). The Shannon diversity index was also not significantly different at 2.11 (SD 0.599) and 2.23 (SD 0.614), respectively (p = 0.32).

We performed weighted principal coordinates analysis and examined their homology, but no significant cluster formation was observed in either the CD14^+^CD11c^+^CD163^low^ fraction or the CD14^+^CD11c^+^CD163^high^ fraction (Fig. 3b).

### The most abundant bacteria present in CD163^low^ and CD163^high^ subsets of CD14^+^CD11c^+^ macrophages from the mucosal lamina propria of Crohn’s disease patients

Tables 3 and 4 depict the OTUs most abundantly present in CD163^low^ and CD163^high^ subsets of CD14^+^CD11c^+^ macrophages in descending order of average read number. The average read number in CD14^+^CD11c^+^ macrophages showed various fluctuations; some were concentrated compared to mucus, and others were diluted compared to mucus. In addition, each subset of CD163^low^ and CD163^high^ showed a different concentration or dilution tendency compared with mucus, with OTUs showing a variety of bacterial distributions. OTU-1: *Acinetobacter;* OTU-2: *Enterobacteriaceae;* and OTU-8: *Pseudomonadaceae* were common to the upper 10 OTUs of the average number of reads of both subsets, and these OTUs were contained in all samples of CD14^+^CD11c^+^ macrophages (Tables 3 and 4). Among them, OTU-1 and OTU-8 tended to concentrate in the CD163^low^ subset. In addition, OTU-17: *Ralstonia* and OTU-18: *Corynebacterium* also showed higher average read number in the CD163^low^ subset than in the CD163^high^ subset and mucus (Table 3). On the other hand, OTU-10: *Proteus* and OTU-15: *Collinsella* tended to be more concentrated in the CD163^high^ subset than in the CD163^low^ subset and mucus, but showed lower mean read number in the CD163^low^ subset than in mucus (Table 4).

**Table 3:** The 10 OTUs with the highest average read number in CD14^+^CD11c^+^CD163^low^ macrophages.

OTUs	Class	Family	Genus	Species	Prevalence (%)	Mean Reads		
						L	H	M
OTU-1	***Gammaproteobacteria***	***Moraxellaceae***	***Acinetobacter***	***Guillouiae***	100	**8754**	**4618**	**1985**
OTU-2	***Gammaproteobacteria***	***Enterobacteriaceae***	Unclassified	Unclassified	100	**4509**	**3249**	**7660**
OTU-8	***Gammaproteobacteria***	***Pseudomonadaceae***	Unclassified	Unclassified	100	**1555**	**605**	130
OTU-4	***Fusobacteriia***	***Fusobacteriaceae***	***Fusobacterium***	Unclassified	91.7	**1464**	**4079**	**3457**
OTU-5	***Erysipelotrichi***	***Eryspielotrichaceae***	94otu1966	97otu2258	83.3	**1129**	**1927**	**3419**
OTU-14	***Clostridia***	***Veillonellaceae***	***Veillonella***	Unclassified	75.0	**598**	228	536
OTU-17	***Betaproteobacteria***	***Oxalobacteraceae***	***Ralstonia***	97otu84368	66.7	**468**	148	149
OTU-23	***Actinobacteria***	***Propionibacteriaceae***	***Propionibacterium***	***acnes***	91.7	**386**	275	113
OTU-18	***Actinobacteria***	***Corynebacteriaceae***	***Corynebacterium***	97otu63787	75.0	**363**	155	11
t-89342	***Clostridia***	***Lachnospiraceae***	Unclassified	Unclassified	41.7	**345**	309	1002

OTU, operational taxonomic unit; L, CD14^+^CD11c^+^CD163^low^; H, CD14^+^CD11c^+^CD163^high^; M, mucus. Prevalence represents the positive rate in all 12 samples of CD14^+^CD11c^+^CD163^low^. In the row showing the mean reads, bold letters indicate that they are within the top ten of each group.

**Table 4:** The 10 OTUs with the highest average read number in CD14^+^CD11c^+^CD163^high^ macrophages.

OTUs	Class	Family	Genus	Species	Prevalence (%)	Mean Reads		
						L	H	M
OTU-1	***Gammaproteobacteria***	***Moraxellaceae***	***Acinetobacter***	***Guillouiae***	100	**8754**	**4618**	**1985**
OTU-4	***Fusobacteriia***	***Fusobacteriaceae***	***Fusobacterium***	Unclassified	100	**1464**	**4079**	**3457**
OTU-2	***Gammaproteobacteria***	***Enterobacteriaceae***	Unclassified	Unclassified	100	**4509**	**3249**	**7660**
OTU-5	***Erysipelotrichi***	***Eryspielotrichaceae***	94otu1966	97otu2258	100	**1129**	**1927**	**3419**
OTU-10	***Gammaproteobacteria***	***Enterobacteriaceae***	***Proteus***	97otu70921	54.5	235	**1074**	501
OTU-9	***Gammaproteobacteria***	***Enterobacteriaceae***	Unclassified	Unclassified	54.5	117	**772**	776
OTU-8	***Gammaproteobacteria***	***Pseudomonadaceae***	Unclassified	Unclassified	100	**1555**	**605**	130
t-86675	***Fusobacteriia***	***Fusobacteriaceae***	***Fusobacterium***	unclassified	45.5	88	**546**	597
OTU-15	***Coriobacteriia***	***Coriobacteriaceae***	***Collinsella***	***aerofaciens***	54.5	59	**418**	352
t-30356	***Clostridia***	***Lachnospiraceae***	***Ruminococcus***	***gnavus***	54.5	74	**407**	692

OTU, operational taxonomic unit; L, CD14^+^CD11c^+^CD163^low^; H, CD14^+^CD11c^+^CD163^high^; M, mucus. Prevalence represents the positive rate in all 11 samples of CD14^+^CD11c^+^CD163^high^. In the row showing the mean reads, bold letters indicate that they are within the top ten of each group.

## Discussion

This study is the first report to comprehensively analyze the bacterial flora in macrophages in the human intestinal lamina propria of Crohn’s disease patients by NGS. First, in Crohn’s disease, we showed that the bacterial flora of CD14^+^CD11c^+^ macrophages in intestinal lamina propria differed in alpha diversity and homology from that of mucus. Furthermore, when OTUs with higher average read number between CD163^low^ and CD163^high^ subsets of CD14^+^CD11c^+^ macrophages were compared, there were OTUs showing different bacterial concentrations or dilutions versus mucus, which suggests bacteria may have different distributions depending on the subset of macrophages.

Dysbiosis is known to occur in the intestinal microbiota of patients with Crohn’s disease^20^, and there are reports on the composition of bacteria present in tissue samples such as the mucosal epithelium and mucosal lamina propria of Crohn’s disease patients compared with stool. In the present study, we clarified the bacterial flora in the macrophages of the intestinal lamina propria from patients with Crohn’s disease in detail by evaluating 16S rRNA gene amplicons. Microbiota in feces is constantly fluctuating even within individuals and can be affected by various means, such as circadian rhythm, diet, medicine, and even the method of obtaining stool samples^21–23^. On the other hand, the bacterial flora present in intestinal macrophages will consist of bacteria invading the intestinal lamina propria beyond barriers such as mucus and intestinal epithelium, and the bacterial flora will also exhibit the ability to escape from macrophagal phagocytosis and digestion processes. A number of genes involved in intracellular bacterial treatment are found in the disease susceptibility gene of Crohn’s disease^24^, and there are reports that in fact macrophages derived from Crohn’s disease patients have reduced ability to treat intracellular bacteria. Therefore, further detailed analysis of the bacteria present inside the macrophage may be the key to clarifying the pathology of Crohn’s disease.

OTU-1: *Acinetobacter*, which was present in all macrophage samples of the mucosal lamina propria of the Crohn’s disease inflamed intestinal tract and was the largest observed by the average read number, was a genus that was significantly increased compared to normal in the 16S rRNA analysis of fecal and mucosal samples of newly developed Crohn’s disease cases in 17 children^25^. In addition, it has been reported that some of the *Acinetobacter* genus showed digestion resistance in a human macrophage cell line and enhanced production of IL-1β, IL-6, and TNF-α by macrophages^26^. OTU-8: *Pseudomonadaceae*, which was also found in all macrophage samples and tended to be enriched with macrophages from mucus, contains *Pseudomonas*. In a study comparing twin patients who developed Crohn’s disease, serum antibody levels against *Pseudomonas* increased significantly compared to non-onset brothers^27^. OTU-18: *Corynebacterium* is another OTU that showed high enrichment in macrophages versus mucus. According to studies in mice, *Corynebacterium*, a resident bacterium in skin, is usually non inflammatory, but increases with a high-fat diet load, which induced IL-17 producing T cells with the coexistence of CD11c^+^ dendritic cells in IL-23 dependent manner, resulting in dermatitis lasting several months^28^. This report also showed an increase in *Proteobacteria* and other *Actinobacteria* along with *Corynebacterium*. The enrichment of bacteria identified in present study may be a mixture of those that can directly affect the pathology and the resulting dysbiosis. This report also suggested that enrichment of certain bacteria might cause human CD11c^+^ cell activation, which leads to Th17 cell induction. *Corynebacterium ulcerans*, one of the species belonging to it, is known as a pathogen showing diphtheria-like symptoms and reportedly has digestion resistance in human macrophages^29^. Thus, bacteria abundantly found in the macrophage samples from this study include those that may have resistance to digestion by macrophages and those that may have immunological responses in patients with Crohn’s disease. It is also interesting that bacteria about which pathogenicity has not been reported are abundantly found inside macrophages. We hope that more detailed phylogenetic and pathogenicity analysis will be verified for these bacteria.

*Proteobacteria* were significantly enriched in the CD14^+^CD11c^+^ macrophage sample of this study, and other gram-negative bacteria such as *Bacteroides* and *Fusobacterium* were also found in the macrophages. CD14 cooperates with TLR4 to pattern recognize the LPS of Gram-negative bacteria. The localization of TLR4 activation causes the activation of receptors on the cell membrane to induce production of inflammatory cytokines through the TIRAP-MyD88 pathway, whereas activation of the receptor on the membrane of endosomes produces type1 interferon through the TRAM-TRIF pathway^30,31^. TLR4 stimulation of the cell membrane surface by pathogenic bacteria reportedly induces the endocytosis of TLR4 while symbiotic bacteria do not stimulate CD14, thereby escaping endocytosis. In addition, the maintenance prostaglandin E2 inhibits the induction of endocytosis of TLR4 to maintain homeostasis^32–34^. We compared the gene expression of CD14^+^CD11c^+^CD163^low^ and CD14^−^CD11c^+^ myeloid cells in intestinal lamina propria from surgically resected intestine of Crohn’s disease patients with normal intestine obtained as surplus intestinal sample of colon cancer^15^. In normal part of cancer samples, the expression of genes that negatively regulate the MyD88-independent TLR signaling pathway and genes that positively regulate prostaglandin E synthesis were enhanced in CD14^+^CD11c^+^CD163^low^ cells compared to CD14^−^CD11c^+^ cells. In contrast, the expression of genes related to the type 1 interferon signaling pathway was enhanced in Crohn’s disease CD14^+^CD11c^+^CD163^low^ cells compared with normal CD14^+^CD11c^+^CD163^low^ cells. Based on the above, there is a possibility that CD14^+^CD11c^+^CD163^low^ macrophages from Crohn’s disease patients may have enhanced activation by LPS stimulation in endosomes, and bacteria involved in this activation process may be obtained this time.

In this study, there was no significant difference between CD163^low^ and CD163^high^ fractions of CD14^+^CD11c^+^ macrophages regarding the diversity and homology of intracellular bacterial flora. However, some OTUs showed contradictory changes in concentration or dilution versus mucus between the CD163^low^ and CD163^high^ subsets, so there may be differences in the ability of the macrophage subset to handle certain intracellular bacteria. It remains to be verified whether the bacteria found in the Crohn’s disease intestinal CD14^+^CD11c^+^ macrophage in this time are actually different in terms of persistence in macrophages and inflammation induction to macrophages.

The present study did not consider comparison between macrophages in the normal intestinal tract and macrophages in non-inflamed part of intestine from Crohn’s disease patients. By combining these analyses with this study’s results, it may be possible to identify the bacterial flora that contribute to the pathology of Crohn’ s disease.
